# Ventricular Noncompaction With Left Ventricular Thrombus: A Case Report

**DOI:** 10.7759/cureus.25605

**Published:** 2022-06-02

**Authors:** Anas Alrefaee, Kyle Wiseman, Ndausung Udongwo, Bharath Sathya, Beverly Demchuk

**Affiliations:** 1 Cardiology, Jersey Shore University Medical Center, Neptune, USA; 2 Internal Medicine, Jersey Shore University Medical Center, Neptune, USA

**Keywords:** ventricular trabeculations, noncompaction cardiomyopathy, noncompaction syndrome, left ventricular thrombus, cardiomyopathy, left ventricular noncompaction cardiomyopathy

## Abstract

Noncompaction cardiomyopathy (NCC) is congenital cardiomyopathy characterized by trabeculations of the left ventricle found on echocardiogram and/or cardiac magnetic resonance imaging (CMRI). This rare disease is associated with thromboembolism and an increased risk of ventricular thrombus formation. We present the case of a 73-year-old female who was admitted for a suspected cerebrovascular accident (CVA), later found on echocardiogram and CMRI to have NCC with left ventricular thrombus. She was started on warfarin indefinitely. We highlight the rarity of this phenomenon as well as the unique questions regarding initiation, length, and choice of therapeutic anticoagulation in the absence of atrial fibrillation in these patients. Consideration of this diagnosis should be made in the absence of other cardioembolic etiologies with prompt management based on available guidelines.

## Introduction

Noncompaction cardiomyopathy (NCC), previously regarded as “spongy myocardium,” is a rare genetic disorder with features of prominent ventricular trabeculations and deep inter-trabecular recesses in either one or both ventricles [1]. Due to its rarity, its prevalence is dependent on reported echocardiographic cases, estimated at between 0.05% and 0.27% [2]. Furthermore, the frequency of thromboembolism in this cohort is ≤38%, and it is more often reported in men [2]. The variable clinical presentations include palpitations, chest pain, shortness of breath, cerebrovascular accidents, and sudden cardiac death. The failure of the loose myocardial meshwork to undergo compaction during fetal development is the proposed pathophysiology of this disease [3-4]. We report a case of a 73-year-old female who presented with uncontrolled high blood pressure, chest pain, and palpitations for several days. Echocardiography revealed findings that were consistent with isolated left ventricular noncompaction cardiomyopathy (LVNC) with concomitant left ventricular thrombus, confirmed with cardiac magnetic resonance imaging (CMRI).

## Case presentation

A 73-year-old female with a past medical history of hypertension, hyperlipidemia, and hypothyroidism presented to the emergency room with multiple complaints. She complained of elevated blood pressure at home (systolic blood pressure >200), bilateral upper and lower extremity paresthesia, chest pressure, and palpitations for four days.

The patient reported chest pressure that started four days prior to admission that was non-exertional. The pain was associated with palpitations, described as “heart racing," and paresthesia of the bilateral hands and feet. The chest pain was described as left-sided pain, pressure-like in quality. She denied any aggravating or alleviating factors. Her chest pain and palpitations resolved prior to her arrival, but her paresthesia persisted. Of note, the patient was active with no known coronary disease, and her blood pressure was well controlled with losartan 50 mg daily and amlodipine 10 mg daily.

Physical examination was remarkable for a blood pressure of 209/88 mmHg and heart rate of 79 bpm. She had an anxious affect but was in no acute distress. A complete heart, lung, and neurological exam was within normal limits.

**Figure 1:**
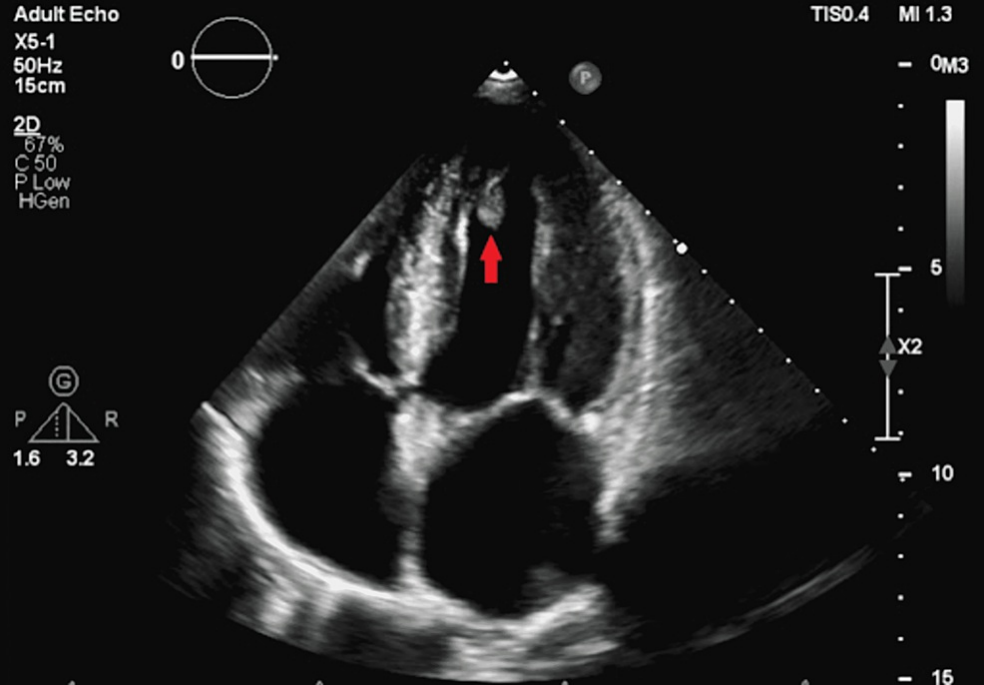
Transthoracic echocardiogram without contrast agent, demonstrating left ventricular apical thrombus (red arrow)

**Figure 2:**
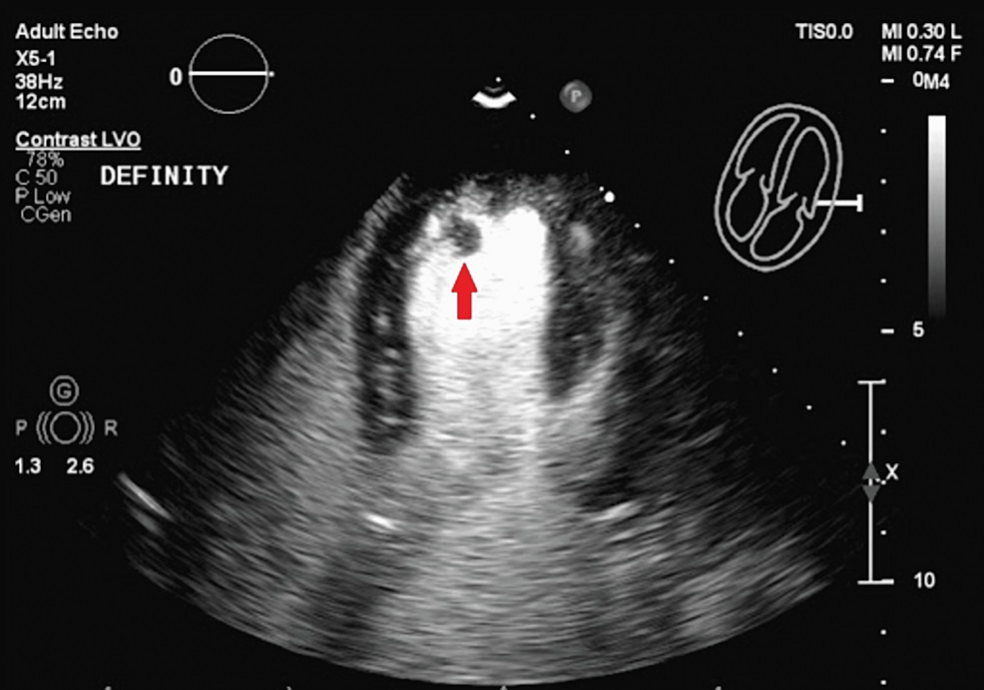
Transthoracic echocardiogram with a contrast agent, demonstrating left ventricular apical thrombus (red arrow)

An electrocardiogram demonstrated normal sinus rhythm and normal axis without any ischemic changes or pathological Q waves. Telemetry for 48 hours did not reveal any arrhythmias. Laboratory results were remarkable for normal serial troponins as well as normal renal and liver function tests. A computed tomography (CT) scan of the head without contrast and MRI of the brain without contrast revealed mild supratentorial white matter chronic microvascular ischemic changes without evidence of acute intracranial processes. CT angiogram of the chest, abdomen, and pelvis ruled out aortic dissection and showed no other abnormalities. An echocardiogram was performed, which revealed a normal ejection fraction with mildly increased left ventricular wall thickness and a mobile echo density in the apex of the left ventricle measuring 1.0 x 0.8 cm (Figures 1-2).

**Figure 3:**
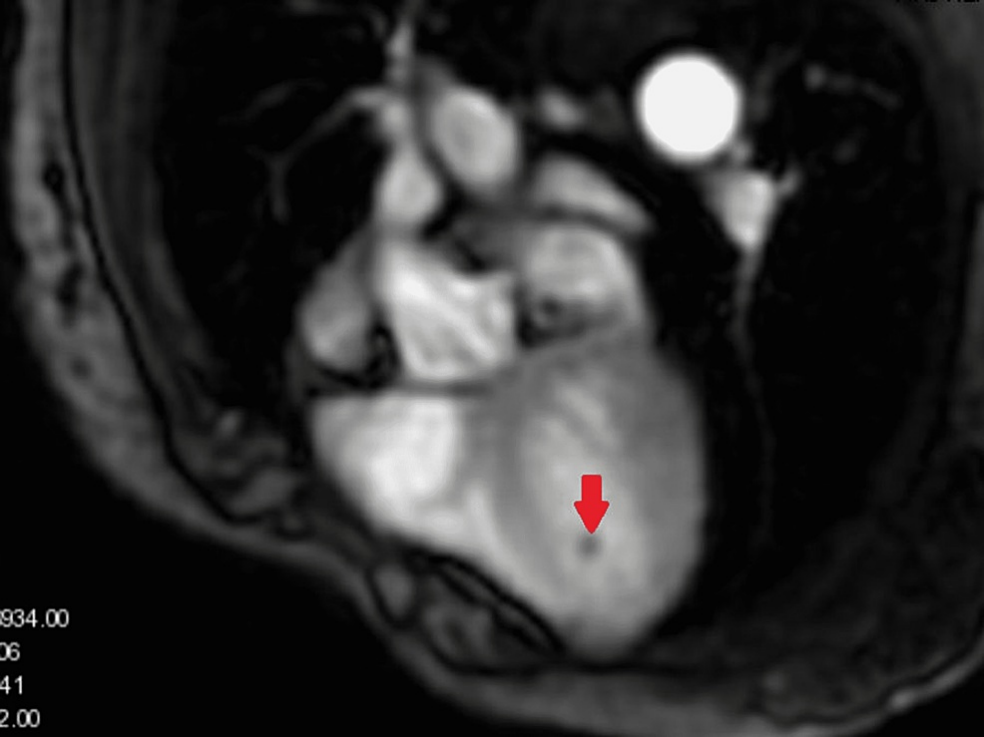
Cardiac MRI demonstrating a filling defect in the left ventricle consistent with a thrombus (red arrow)

**Figure 4:**
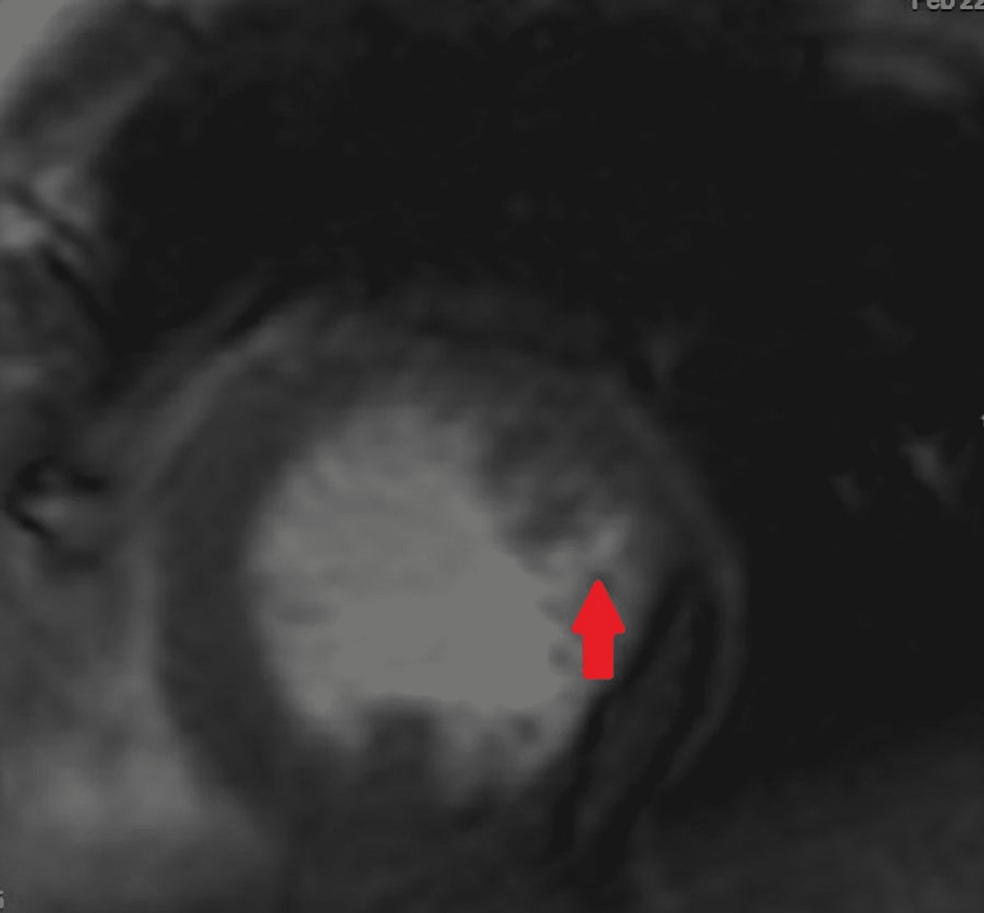
Cardiac MRI demonstrating left ventricular non-compacted myocardium (red arrow)

Prominent trabeculations were also noted that were suggestive of noncompaction cardiomyopathy. Cardiac MRI demonstrated hypertrabeculation of the left ventricle with a 2.37 ratio of non-compacted to compacted myocardium. There was no late gadolinium enhancement suggestive of myocardial infarction or fibrosis. The mass located in the apical region of the left ventricle was isointense to the myocardium on T1W imaging and heterogeneously hyperintense on T2W imaging. The mass took up less contrast than the myocardium on perfusion imaging, and peripheral enhancement on late gadolinium enhancement was consistent with an organized thrombus (Figures 3-4).

The patient had negative biomarkers for cancer and no evidence of metastatic disease on CT of the chest, abdomen, and pelvis. She was started on an unfractionated heparin drip as a bridge to warfarin therapy and was discharged on warfarin 10 mg daily with an international normalized ratio (INR) goal of 2-3. She will follow up in the office for a repeat echocardiogram four to six weeks from discharge. The plan is to continue warfarin therapy indefinitely. The patient will have regular follow-ups with her cardiologist for arrhythmia screening and monitoring of her left ventricular function. The available literature indicates that warfarin will be effective at preventing further embolic events with a resolution of left ventricular thrombus on a follow-up echocardiogram.

## Discussion

Noncompaction cardiomyopathy (NCC) is rare genetic cardiomyopathy [2] first described in 1932 and characterized by excess trabeculae in the endocardium, affecting one or both ventricles [2,5-6]. Prevalence estimates range from 0.01% to 0.27% among patients who were referred for an echocardiogram for other workups [2,5]. In reference to the entire population, estimates range from 0.014% to 1.3%, with males affected more than females. It is often an autosomal dominant inheritance pattern with defects in genes coding for sarcomere, cytoskeleton, and mitochondrial proteins [5].

The mechanism of NCC is thought to be due to a failure of “compaction” of the myocardium during embryogenesis [5]. The diagnosis is established most commonly via transthoracic echocardiogram and cardiac magnetic resonance imaging [2], with echocardiogram showing a ratio of greater than 2.0 between the end-systolic thickness of the compacted vs noncompacted tissue [7]. Recent reports indicate high sensitivity and specificity using a ratio of >2.3 [7]. Low-dose cardiac CTs and left ventriculograms have been useful in other cases undergoing ischemic evaluation; however, no gold standard for diagnosis yet exists [2,5].

NCC is often associated with cardiac structural abnormalities, and isolated NCC can occur as well [5]. On imaging, the subendocardial layer of the left ventricle remains more trabeculated than normal with some studies suggesting a pathogenic role of ischemic damage to the endocardium, as evidenced by fibrotic changes on cardiac-MRI [4]. The most common cardiac manifestations include thromboembolism and cerebral embolism [5]. Four case series manuscripts were noted in a 2015 literature review to have incidences of left ventricular thrombus ranging from 0% to 25%, cerebral embolism ranging from 0% to 25% [5], and findings on 2D echo showing LV thrombus in many of these patients [8-10]. An additional report of a 34-patient study revealed thromboembolic events present in 24% of the patients [10-11]. However, the independent association between NCC and thrombus or thromboembolism is still not well-established. One study indicated a 1-2% stroke risk with thromboembolism risks ranging from 21% to 38% [11].

The risk of left ventricular thrombus and the mortality associated with stroke in isolated left ventricular noncompaction cardiomyopathy is unknown due to the rarity of the disease and the lack of prospective trials [2]. However, these events are more common in adults [4]. The mechanism is thought to be propagated by the deep recesses present in the trabeculae of the ventricle, leading to clot formation [10], with reduced LV function contributing as well [6].

The 2019 Heart Rhythm Society (HRS) expert consensus recommends anticoagulation in patients with NCC and atrial fibrillation, patients with NCC who have previously experienced a stroke, or NCC patients who have a known LV thrombus [2,6]. The consensus also states that anticoagulation may be appropriate in patients with NCC and left ventricular dysfunction, class of recommendations (COR) IIb, level of evidence (LOE) B-NR [6]. A recent literature review suggests that aspirin alone is not enough for stroke prevention in these patients [2]. Two case reports suggest that direct oral anticoagulants (DOACS) may be appropriate as well [12-13], especially given the findings of complete resolution of LV thrombus in non-NCC patients with equal efficacy using DOAC therapy [11,14]. Other studies argue that warfarin is superior to DOACs in preventing embolism in patients with evidence of LV thrombus [15]. However, dosing of the DOACs and bleeding events were not reported, weakening the evidence and establishing the need for further studies [2]. Studies have suggested using a “CHADS” (congestive heart failure; hypertension, age over 75 years, diabetes mellitus, and a previous history of stroke) score, similar to that used to stratify stroke risk in patients with atrial fibrillation and in patients with NCC as well regardless of whether they have atrial fibrillation or not [11,16].

The initiation of warfarin for secondary prevention of stroke in NCC patients is a class 2a recommendation [11]; however, the length of anticoagulation therapy in these patients has not been established. Recommendations for individualized decision-making are noted when deciding between antiplatelet versus anticoagulant treatment in patients with reduced ejection fraction (EF) without ventricular thrombus in the absence of atrial fibrillation [11]. If using warfarin, a targeted INR range of 2.0-3.0 has been proposed in patients with reduced LV function [11]. The mortality of NCC is strongly associated with cardiac risk factors, such as arrhythmias and ventricular dysfunction, thus warranting close monitoring [4].

Follow-up consists of arrhythmia screenings, including electrophysiology (EP) evaluation if needed, especially given the reported incidences of endocardial and epicardial foci of arrhythmias [11]. These patients also benefit from genetic counseling. In the setting of systolic and/or diastolic dysfunction, guideline-directed medical therapy is appropriate with consideration of implantable cardioverter-defibrillator (ICD) devices or transplants if indicated [11]. The outcomes of transplant patients have yet to be established.

## Conclusions

The incidence and mechanism of LV thrombus formation in this group of patients vary between studies but can lead to debilitating sequelae, including cerebrovascular accident (CVA). Imaging tests, particularly 2D-echocardiography and CMRI play a pivotal role in the diagnosis and management of this unusual disease. However, more studies are needed to establish the choice and duration of anticoagulants, including the efficacy and safety of DOACs in left ventricular noncompaction cardiomyopathy (LVNC) patients.
